# Comparison of computed tomography and high-field (3.0 T) magnetic resonance imaging of age-related variances in selected equine maxillary cheek teeth and adjacent tissues

**DOI:** 10.1186/s12917-017-1200-7

**Published:** 2017-09-06

**Authors:** Christin Schoppe, Maren Hellige, Karl Rohn, Bernhard Ohnesorge, Astrid Bienert-Zeit

**Affiliations:** 1 Clinic for Horses, University of Veterinary Medicine Hannover, Foundation, Buenteweg 9, 30559 Hannover, Germany; 2 Institute for Veterinary Biometry and Epidemiology, University of Veterinary Medicine Hannover, Foundation, Buenteweg 2, 30559 Hannover, Germany

**Keywords:** Horse, Ct, 3 tesla, High-field MRI, Equine maxillary cheek teeth, Age-related variances, Endodontic disease, Periodontal

## Abstract

**Background:**

Modern imaging techniques such as computed tomography (CT) and magnetic resonance imaging (MRI) have the advantage of producing images without superimposition. Whilst CT is a well-established technique for dental diagnostics, MRI examinations are rarely used for the evaluation of dental diseases in horses. Regarding equine endodontic therapies which are increasingly implemented, MRI could help to portray changes of the periodontal ligament and display gross pulpar anatomy. Knowledge of age-related changes is essential for diagnosis, as cheek teeth and surrounding structures alter with increasing age. The aim of the present study was to highlight the advantages of CT and MRI regarding age-related changes in selected equine cheek teeth and their adjacent structures.

**Results:**

The CT and MRI appearances of the maxillary 08 s and 09 s and adjacent structures were described by evaluation of post-mortem examinations of nine horses of different ages (Group A: <6 years, B: 6–15 years, C: ≥16 years). Most of the tissues selected were imaged accurately with MRI and CT. Magnetic resonance imaging gives an excellent depiction of soft endo- and periodontal units, and CT of hard dental and bony tissues. Negative correlation between dental age and pulpar sizes was found: 71.3% of the changes in pulp dimensions can be explained by teeth aging. Pulpar sizes ranged from 14.3 to 1.3 mm and were significantly smaller in older horses (*p* < 0.05). A common pulp chamber was present in 33% of the teeth with a mean dental age of 2.25 years. Ninety-four percent of the 08 and 09 alveoli of all groups were in direct contact with the maxillary sinus. An age-related regression was found (R^2^ = 0.88) for the distance between alveoli and the infraorbital canal.

**Conclusions:**

The present study provides information about the dental and periodontal age-related morphology and its visibility using different imaging techniques. These results aid in evaluating diagnostic images and in deciding which is the superior imaging modality for clinical cases.

## Background

Radiography has been the primary imaging modality historically for evaluating the heads, paranasal sinuses, and dental and periodontal structures [1]. Modern imaging techniques, such as computed tomography (CT) and magnetic resonance imaging (MRI), offer the benefit of producing three-dimensional (3D) images without superimposition and the possibility of multiplanar reconstructions (MPRs). Both CT and MRI are viable imaging modalities for evaluating the head in human [2–4] and veterinary medicine [5–10]. The well-established CT provides a good spatial resolution and excellent delineation of bony tissue [11], but is limited due to its inability to visualise pulpar and periodontal tissue with detailed resolution [12]. Whilst MRI, with its detailed depiction of soft tissues, has already become a valuable tool for diagnosing oro-dental diseases in humans [13–16], it is rarely used in equine dentistry [17]. Applications reported in the human medical sector include the evaluation of the temporomandibular joint [18, 19] and the nerve channels [20–22], as well as orthodontic utilisation for the assessment of impacted teeth [23], dental pulps or caries diagnosis [13] and apical periodontitis [24]. In accordance with these findings, a 1.5 Tesla MRI was used to portray physiological and pathological cheek teeth [17, 25] and their surrounding structures in equine dentistry [26]. Good MR image acquisition holds enormous potential for radiation-free diagnostic orthodontic workups [27], especially in times when endodontic procedures in conjunction with pulp infection become more common in equine clinical practice [28, 29].

Knowledge of age-related changes and their presentation in 3D imaging modalities is essential for diagnosis and surgical planning since the anatomy of equine hypsodont cheek teeth and neighbouring structures alters with increasing age. While CT, cone-beam CT and 3 Tesla-MRI dental imaging have been directly compared in humans [14], little is known about the dental head-to-head comparison of CT and high-field MRI in different age stages of equine patients.

The purpose of this study was to analyse the visualisation of anatomical landmarks (endo-, periodontal and adjacent structures) and their changes with increasing age in CT and MR images, aiming to highlight the best imaging technique for each structure at different ages. The maxillary 08 s and 09 s were chosen for the examinations because they belong to the cheek teeth with the most frequent pathologies [30].

## Methods

### Specimens

Nine horses of different breeds (eight warmbloods, one standardbred) were examined to acquire CT and high-field MRI scans of selected equine cheek teeth, their periodontal tissues and adjacent structures. The horses were clinic-owned (TiHo Hannover, Clinic for horses, Germany). The study population was divided into three groups according to age classes: Group A “young” (2–5 years), B “middle-aged” (6–15 years) and C “old to senile” (≥ 16 years), each with *n* = 3 horses. The age of the selected horses ranged from 2.4 to 21.7 years (median 8.5 years). All horses were subjected to euthanasia on human grounds for non-dental reasons and for health purposes not related to this study. When evaluating the CT and MR images, the Triadan system was used for numbering the cheek teeth [31]. A total of 36 maxillary cheek teeth were examined. The sample pool included eighteen Triadan 08 s and eighteen Triadan 09 s. Owing to eruption time, dental age was determined by subtracting the earliest possible eruption age of the tooth from the age of the horse [32]. An eruption time of 3.5 years for the 08 s and six months for the 09 s was assumed [33]. The population included selected teeth between 0.9 and 20.95 years (median age 7.95 years).

### Diagnostic imaging techniques and image evaluation

The imaging processes were carried out at the University of Veterinary Medicine Hannover, Foundation. Images were taken within six hours after euthanasia. The horses were first placed on a stationary CT table in right lateral recumbency and afterwards in dorsal recumbency on a non-stationary MRI table. Dorsal and transversal scan series of the head were acquired (Table 1). The orientation for transverse planes was perpendicular and dorsal planes were orientated parallel to the hard palate.

**Table 1:** Imaging techniques and settings

Imaging technique	Sequence	Orientation	Matrix	TR (ms)	TE (ms)	ST (mm)
MRI	T1w	3D	1024	8.5	3.9	0.9
	T2w	transverse	1024	4500	80	3.1
	T2w	dorsal	1024	3000	80	4
	PDw	dorsal	1024	6400	30	4
	STIR	transverse	960	8872	30	3
CT	Helical Scan	transverse	1024			1.5

*TE* echo time**,** *TR* time to repetition**,** *ST* slice thickness

The CT image acquisition was performed with a Brilliance™ CT – Big Bore Oncology Scanner (Philips Medical System, Best, The Netherlands). Images of the head were acquired using a modified dental scanning protocol with 140 kV and 300 mAs. A Philips Achieva™ 3.0TX-Series® was used for MRI acquisition. Surface coils (Philips SENSETM FlexM® and Philips SENSETM FlexL®) were positioned around the region of interest (ROI), between the rostral margin of the facial crest and the orbital cavity. The MRI sequences obtained were: T1 weighted (T1w), T2 weighted (T2w), proton density weighted (PDw) and PD fat-suppressed short-tau-inversion-recovery (STIR) images.

### Visualisation and measurements

The CT and MR images were examined and visualisation of the dental and adjacent structures depicted was performed. All examinations were observed by M.H., an experienced radiologist, A.B.-Z., Diplomate EVDC (Equine), and C.S., a trained veterinarian.

Anatomical landmarks (endo-, periodontal and adjacent structures) and their visualisation in CT and MRI were described (Tables 2 and 3).

**Table 2:** Anatomical landmarks of dental structures depicted in MRI and CT referring to imaging advantages

Anatomical region		CT	MRI
Endodontic system	Dental sac	Low attenuated tissue surrounding the reserve crown of growing cheek teeth (transverse)	High signal intensity, marked off by the alveolar bone with low signal intensity (T2w and STIR, both transverse)*
	Common pulp cavity	Low attenuated pulp tissue in young maxillary teeth (transverse)	Hyperintense soft tissue, present in young cheek teeth (T2w transverse, PDw dorsal)*
	Pulps	Generally five pulp horns with very low density visible, moderate to well distinguishable from the hyperdense hard dental tissue; Pulp: −350 to 500 HU (transverse)	Generally five hyperintense pulp horns visible Very good distinction from the hard dental tissue (T2w, STIR transverse; PDw dorsal)*, especially in young cheek teeth
Anatomical crown	Tooth root	Isodense root (transverse)*	Isointense: with good alignment of the MRI scan, well demarcated structure (T2w transverse)
Clinical crown	Hard dental tissue, distinction to the oral cavity	Dentine, cement and enamel: distinction possible through the different grade of attenuation; Hyperdense enamel and dentine; slightly less opaque cement (transverse, dorsal)*	Dentine, cement and enamel: signal free, not distinguishable (T2w and STIR transverse) Distinction to the oral cavity not displayable, only visible where tongue with moderate signal intensity is in contact with the dental crown or saliva surrounds the tooth (T2w and STIR, transverse)

*superior imaging modality by comparison of the respective structure in CT and MR images (in brackets: the best imaging modalities and section planes)

**Table 3:** Anatomical landmarks of periodontal and adjacent structures depicted in MRI and CT referring to imaging advantages

Anatomical region		CT	MRI
Periodontal apparatus	Periodontal space: Periodontal ligament (PDL)	Slightly blurry, isodense gap between hard dental tissue and alveolar bone, moderate distinction from hard dental and bony tissue (transverse, dorsal)	Hyperintense gap, good distinction of hard dental tissue and alveolar bone* (T2w and PDw dorsal)
	Alveolar bone	Hyperdense bone* (transverse, dorsal)	Hypointense cortical bone, marked off by the PDL (T2w and STIR transverse)
Maxillary sinus	Mucosa and cortical bone	Respiratory epithelium: not visible; sinus: air-filled and hypodense; hyperdense thin-walled cortical bone* (transverse)	Signal free cortical bone, delineated by a hyperintense line of mucosa* (T2w and STIR transverse)
IOC	Soft tissue inside and bony structures	Soft tissue inside the canal: low attenuated IOC*: high attenuated, very well distinguishable (transverse)	IOC: signal free, becomes evident through the bright mucosa covering the bony surface of the maxillary sinus (T2w and STIR transverse) Soft tissue*: inhomogeneous, high to moderate signal, intense structures inside: nerve with moderate signal intensity, blood vessels with high or low signal intensity (T2w and STIR transverse)

*superior imaging modality by comparison of the respective structure in CT and MR images (in brackets: the best imaging modalities and section planes)

**Fig. 1:**
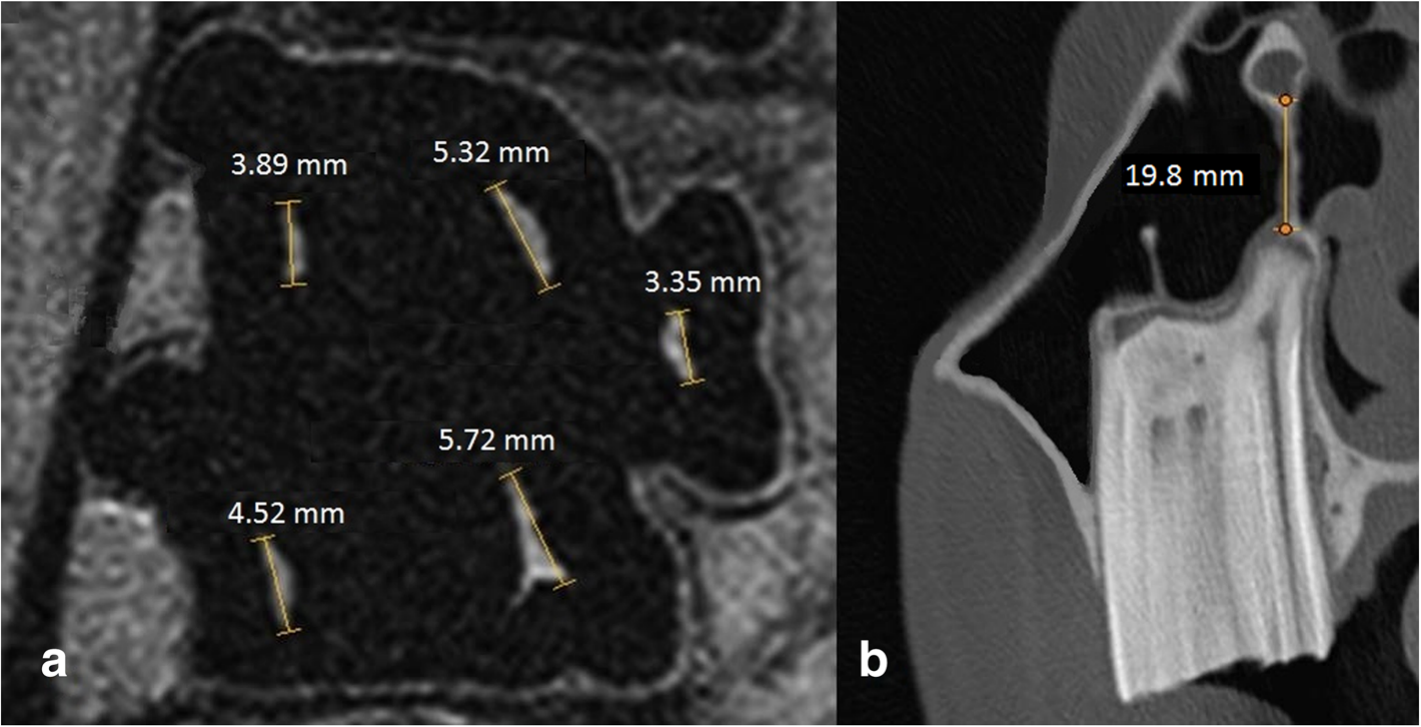
Measurements of pulpar sizes and distance between alveolus and infraorbital canal (IOC). Pulp dimensions of an 8.5-year-old tooth (108, dorsal plane) in PDw magnetic resonance images (**a**); distance between the alveolar socket and the IOC in a 6-year-old tooth (209, transversal plane) using CT imaging (**b**)

Special attention was paid to age-related changes. The positional relations between the dental roots and the floor of the paranasal sinuses were described. Pulpar sizes and the positional relation between the dental alveoli and the infraorbital canal (IOC) were portrayed. Each pulp was measured in the mid-tooth section of the cheek tooth selected (Fig. 1a) after each tooth’s half-length was determined in the transverse MRI scans. The distance between the dental alveoli and the IOC was portrayed in transverse sections of the CT images. Therefore, the line between the dental alveolus above the pulp position five and the inner border of the IOC was measured (Fig. 1b). Pulpar sizes and the distance between the IOC and the dental alveoli were measured by one of the examiners (C.S.) using easyVET image editor (easy Vet, IFS Informationssysteme GmbH, Hannover, Germany). All measurements were repeated three times under the same conditions and the median value for each pulp or each distance (between the dental alveoli and IOC) was obtained. Moreover, the IOC’s shape was assessed.

### Statistical analysis

Data were collected on spreadsheets (Excel® 2010, Microsoft® Corporation Redmond, Washington, USA). SAS® software (SAS Institute, Cary North Carolina, USA) was used for statistical analysis and GraphPad Software, Inc.® (La Jolla, California, USA) was used for graphical and statistical representations. Data were tested for normal distribution with the Kolmogorov-Smirnov test. Correlation between dental ages and pulp sizes or alveolar-infraorbital distance was tested by Spearman’s rho. A Wilcoxon matched pairs signed rank test was chosen to calculate whether there were significant differences in the pulp sizes or alveolar-infraorbital distances between both sides of the maxilla. The Kruskal-Wallis test and Dunn’s multiple comparison test were performed to validate significant differences of pulp dimensions between different age groups. A *p* value <0.05 was considered statistically significant.

## Results

Nine horses were examined immediately after euthanasia. Images of the entire head were acquired by CT and MRI settings were adapted to accomplish maximum image quality within a reasonable examination time. The field of view (FOV) in the MRI scans was limited from a transverse line caudal to the 09 maxillary teeth to the rostral end of the 08 upper cheek teeth. It ranged from 180 to 250 mm in dorsally orientated sequences and from 180 to 220 mm in transversely orientated MR images. The duration of the CT scans was short (mean ± SD, 2 ± 5 min); the MRI examination took between 80 and 98 min (mean ± SD, 89 ± 9 min). While dorsally orientated MR image acquisition took between 10 (T2w) and 16 min (PDw), transversal scan duration took between 18 (T2w) and 24 min (STIR). The T1w 3D image scan for MPR lasted approximately 14 min.

### Imaging modalities

Anatomical landmarks and imaging advantages comparing CT and MR images regarding the maxillary 08 s and 09 s and their adjacent structures were evaluated and summarised in Tables 2 and 3. Acquisition by CT proved to be the best imaging technique for the representation of the bony non-dental structures (alveolar bone, cortical and cancellous maxillary bone and the IOC). These bony structures were well delineated against the low attenuated soft tissues. Dental enamel, dentine and cementum were also detected as structures of specific density, marked with different greyscales by CT imaging, but the differentiation between them was not as clear as the line between the alveolar bone and the periodontal space due to their different opacity. Regarding the endodontic system, CT images revealed not only hypodense pulp horns and chambers, but also hypodense funnel-like infundibula. The infundibula could not be detected on the MR images. Marked hypoattenuating stripes were visible adjacent to the infundibula in some of the CT slices, that could be determined as gas. Compared with slightly blurred pulpar CT imaging, the pulp tissue appeared as bright, sharp, hyperintense pulp on the MR images. Other soft tissues, such as the periodontal ligament, respiratory mucosa and the infraorbital nerve, were also better visualised with MRI compared to CT images.

### Age-related pulpar sizes

A total of 177 dental pulp measurements were acquired in the upper 08 s and 09 s on dorsally oriented PDw sequences of the MR images. Calculated dental ages for the equine age groups are documented in Table 4. One of the pulp horns was not visible (always pulp 5) in each of three older cheek teeth of the age group B and C (a 10-year-old 209 and a 15.75-year-old 109 and 209). A differentiation between the hypointense hard dental and the normally hyperintense soft pulp tissue could not be performed in any of these MRI sequences because both tissues appeared hypointense in these teeth. Thus, three pulps were excluded from the pulp measurements. The cheek teeth in all other maxillary 08 s and 09 s contained five pulp horns, named pulp 1 to pulp 5 (P1-P5) [34].

**Table 4:** Calculated dental age of the upper 08 s and 09 s related to the horses’ age

Age group	Horses’ age in years	Dental age in years: 08 s (n)	Dental age in years: 09 s (n)
A	< 6	< 2.5 (6)	< 5.5 (6)
B	6–15	2.5–12.5 (6)	5.5–15.5 (6)
C	≥ 16	≥ 12.5 (6)	≥ 15.5 (6)

**Fig. 2:**
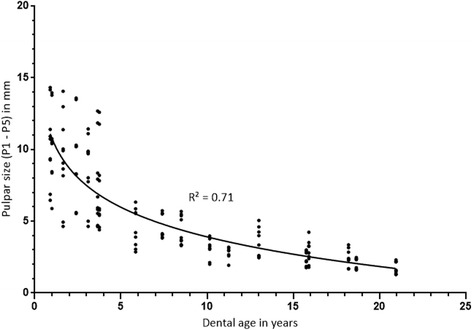
Semi-logarithmic scale of age-related pulp horn sizes (pulp 1 - pulp 5). Pulps were measured in the mid-tooth section of the maxillary 08 s and 09 s in MR image scans (PDw, dorsal orientation); P1-P5: Pulp horn 1–5

**Fig. 3:**
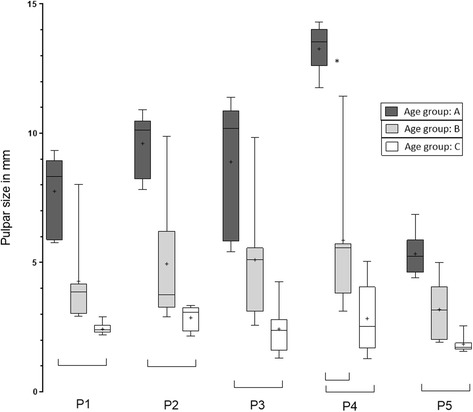
Pulp measurements of MR images in different age groups. Examination was performed with PDw scans. Horizontal whiskers represent statistically significant differences in size between pulp horns. Boxes represent the interquartile range, vertical whiskers the range and “+” show mean in all boxplots; P1-P5: pulp horn 1–5

**Fig. 4:**
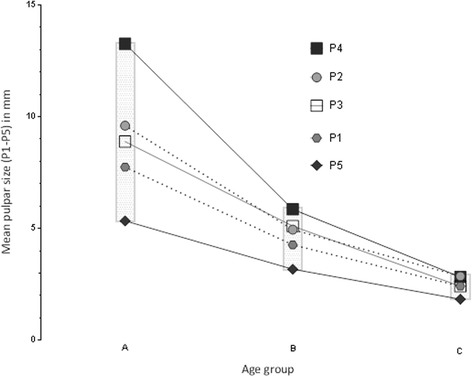
Different mean reduction of each pulp between different age groups. Mean pulpar sizes presented in different age groups, measured in dorsally orientated MRI scans. Boxes show the range between the mean largest and smallest pulp in each age group. P1 – P5 present pulp horn 1 to pulp horn 5

Negative correlation between dental age and pulpar dimensions was found (*r* = −0.9). Pulpar size decrease can be explained by dental aging in 71% of the teeth examined: the older the selected cheek teeth of clinically healthy horses became, the smaller the measurements of the pulps (Fig. 2). The sizes of all five pulps varied with age between 14.31 and 1.7 mm for the 08 s and between 14.05 and 1.3 mm for the 09 s. Other pulpar sizes are documented in Table 5. Comparing the pulp horns, pulp 4 was the largest and pulp 5 was the smallest, except for two teeth (Triadan 109) of age group C, where pulp 3 showed the smallest size. Whilst young teeth showed wide pulpar size variations, the range of size differences regarding all pulps became smaller with increasing age; this is visualised by the boxes in Fig. 4. There was no significant difference in the pulpar dimensions between both sides of the maxilla. Significant differences were measurable for the same pulp horn position in all teeth by comparing of the pulp sizes between age group A and C. Pulp 4 also showed a significantly smaller pulpar size between group A and B (Fig. 3). The percentage reduction of mean pulpar size comparing both age classes was between 40 (for all P5) and 56% (for all P4). Pulpar size decrease was smaller considering the same comparisons between the pulps of group B and C, with values ranging from 42 (P3, P2) to 53% (Fig. 4).

**Table 5:** Pulp parameters. MRI measurements were conducted in the mid-tooth section of the upper maxillary 08 s and 09 s. Results show pulp parameters independent of age; n = number of pulp horns; P1-P5: Pulp 1–5

	P1	P2	P3	P4	P5
n	35	36	34	36	36
Mean (mm)	4.89	5.81	5.57	7.32	3.45
s.d. (mm)	2.59	3.26	3.42	4.78	1.65
Median (mm)	3.89	3.75	5.24	5.67	3.17
Range (mm)	7.15	8.76	10.09	13.02	5.30
x_min_ – x_max_ (mm)	2.19–9.34	2.16–0.91	1.3–11.39	1.28–14.31	1.57–6.87

### Common pulp chambers

The common pulp chambers (CPCs) were evaluated in dorsally orientated PDW sequences of MR images. A CPC was encountered in all cheek teeth of age group A (*n* = 12). The median dental age for these cheek teeth was 2.025 years and the maximum age was 3.75 years. Cheek teeth of the other age groups showed no CPC.

### Positional relations between the maxillary sinus and the dental alveoli

Only two dental alveoli (upper 08 s of age group A) out of all 36 cheek teeth were not located below the maxillary sinus. Positional relations of the structures could be seen in both imaging modalities. Whilst CT images highlighted the bony alveoli, the MRI scans delineated the differentiation of sinus and alveolus through the hyperintense periodontal ligament (PDL) and the sinus mucosa. Eight of the 34 teeth roots located within the boundaries of the sinus cavity were only located below the maxillary sinus with their caudal aspect (08 s), including pulp horn 2 and 4. Fifty percent of these teeth (*n* = 4) partly contacting the sinus floor contained a CPC. The percentage of teeth that were positioned below the maxillary sinus with all five pulps (*n* = 26) consists of 08 s (*n* = 8) and 09 s (*n* = 18).

### Age-related changes of the infraorbital canal and its distance to the dental alveoli

**Fig. 5:**
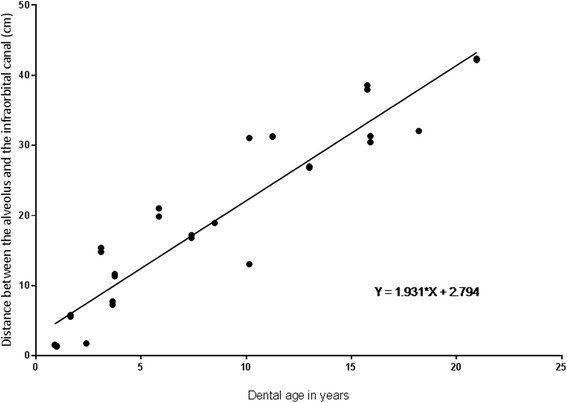
Age-related distance between selected cheek teeth (*n* = 36) and the IOC. Scatterplot of the distance between dental alveoli of 08 s and 09 s and the bony IOC measured in transverse CT images

Age-related changes of the IOC were analysed in transverse CT scans showing the positional relation between tooth and bony canal, on the one hand, and the canals’ shape, on the other (Fig. 1b). Where the bony IOC was not in contact with the alveolar bone, it was oval-shaped and held in position by a free bone bridge. If the cheek tooth alveolus and the IOC were in close contact, the bony canal was irregularly shaped. Age-related positional changes of the IOC could be measured (Fig. 5). Two teeth roots (both 08 s) of the youngest horse in group A showed direct contact with the IOC, but the distance between dental alveolus and IOC increased with dental age. The increase in distance in 88% could be explained by dental aging (R^2^ = 0.88). There was no significant difference between both sides of the maxilla.

## Discussion

### Imaging modalities

As detailed evaluation of the internal and periodontal structures of the cheek teeth is important in deciding on treatment when pulpar or apical infection occurs [35], CT and MR images have been acquired in the present study, aiming to highlight the best imaging modality for each structure. The overview of the two techniques on the same head allows a direct comparison of the potential of both. There are several studies published in equine medicine describing the qualities of CT or MRI for the diagnosis of head pathologies without comparison of both techniques [1–6] and with lower MRI field strength of 0.5 to 1.5 T [17, 25, 36].

The image quality of MRI in the present study was comparable to that of CT, but was better for bony tissues in CT and for soft tissues in MRI. The present results correspond with the findings of Gerlach et al. [36] and Kaminsky et al. [10], who proved the excellent quality of MRI in portraying the dental pulp, PDL, bone-marrow, gingiva, facial soft tissue, sinus mucosa, infraorbital nerve and vessels, owing to a high water content and hydrogen atoms. There was no detectable MRI signal from hard dental tissues, cortical bone, lamina dura and the IOC, whereas these structures could be visualised with good detail resolution by CT acquisition. The best MR image quality in the present study could be achieved with T2w scans for an anatomical overview and with PDw or STIR scans for more detailed images; this is in line with the findings of Kraft and Gavin [37], where T2w, STIR and PDw scans appeared superior to T1w scans in all images evaluated. This is related to the thinner slice thickness of the 3D T1w sequences, which are more often affected by artefacts compared to the transversal and dorsal orientated sequences of the STIR, PD and T2 weighted images with thicker slices.

Compared to low-field tomography, 3.0 T MRI has a higher signal-to-noise ratio, leading to better resolution. Consequently, sequences can be taken with a lower recording time and equal image quality compared to low-field MRI. The time for general anaesthesia for patients can be shortened and anaesthesia risk is reduced [38, 39]. The time required for imaging examination in the present study differed greatly between MRI and CT and between the different MRI scans. The CT was around 18 times faster than the entire MRI examination, due to the different imaging techniques and various MRI scans. The long MRI examination times were chosen to acquire images of excellent quality in the current study. The number of image alignments (transverse, dorsally orientated), resolution, or FOV might be decreased for clinical use to reduce scan time.

Due to the differences in the alignment of teeth [40], the MR images obtained could include artefacts, because image alignment was chosen for the entire skull and not for each tooth. Finally, some pulps and PDLs appeared blurry only due to image angulation (T2w, PDw, STIR) in the current study, that might be mistaken for pathology [17]. The alignment for each tooth would take more time and anaesthetic risk in clinical patients would increase due to longer examination times [41]. T1w images with 3D datasets offer an exception: through MPR, this technique provides the possibility of producing image planes in alignment with the teeth depicted. The time for image acquisition in T1w sequences is prolonged in high-field MRI, as T1 relaxation time is longer [38]. Therefore, a short acquisition time for T1w images, as was applied in the current study, is always accompanied by worse image quality compared to the T2w scans.

In contrast to MRI, CT provides the possibility of different angulations through subsequent MPR and, thus, the potential to evaluate the dental and periodontal changes in alignment of every single tooth. Nevertheless, CT also shows limitations, as it is less useful in identifying early changes in the pulp [42] due to its inability to visualise soft tissues as excellently as T2w or PDw images.

### Selected cheek teeth

As described in the literature, dental pathologies such as apical infections, dental fractures and infundibular caries occur predominantly in certain maxillary cheek teeth [43]. The upper 08 s and 09 s show clinical signs most frequently [44–46] and, therefore, they were chosen for examination in the current study.

### Selected age groups

The planning of surgical procedures in cases of dental and associated pathologies requires accurate anatomical knowledge and imaging of the cheek teeth and adjacent structures, which are illustrated in this study. Selected equine groups of different ages were chosen in our study as dental changes can be influenced by morphological, functional and mechanical changes, which occur with increasing age [47]: Equine cheek teeth show second dentition and real longitudinal growth up to the horse’s age of five years (Group A in the current study); afterwards, the tooth length decreases as teeth are pulled out of the alveoli in the oral direction and the cheek teeth abrasion continues (Group B in our study: 6–15 years) [48, 49]. In older horses (Group C from an age of 15 years onwards), cheek teeth have an exposition to higher dental forces [50]. All these aspects might influence changes of dental structures (e.g. pulpar size) or positional relations between dental and periodontal structures, which were examined in the current study.

Regarding the preselected horse groups (resulting in preselected dental age groups), the present evaluations should be treated with considerable caution: due to the small size of the study population, age distribution and image interpretation might not comply with the entire equine population.

### Age-related variances

#### Common pulp chamber

It can be assumed that CPCs are more common in younger teeth [51], which is in line with the current results, as CPCs were displayed in all teeth evaluated of age group A with a mean dental age of 2.25 years. Dacre et al. [32] located a CPC in teeth with a mean dental age of 2 years (via microscopic examination), whereas Kopke et al. [52] stated the maxillary CPC to be evident in teeth with a mean dental age of 4 years (in high resolution micro-CT scans). While CT [28] and MRI studies [25] observed a CPC in cheek teeth up to 6 years and with micro-CT even up the dental age of 9 years [52] in the present study, the oldest teeth showing a CPC had a dental age of 3.75 years. The difference in results could be caused by the different imaging techniques, imaging settings or measuring techniques. More precise results could probably be achieved by micro-CT scans and histological investigations.

#### Pulps

Anatomical examinations of the endodontic system by Baker [53] and Dacre et al. [32], as well as MRI [25] and CT [28] imaging studies revealed a general pattern of five pulp horns in the maxillary 08 s and 09 s. These findings comply with the results of the current study, where five main pulp horns (P1-P5) were visible in 33 cheek teeth in the CT and MR images. Three out of all 36 cheek teeth evaluated showed only four pulps.

Contrary to the histological findings of Shaw et al. [54], which displayed an increase in pulpar sizes between 3.5- and 7-year-old teeth, the MRI measurements in the current study showed a continual reduction in size with age. This is consistent with MRI [25] and CT [28] studies demonstrating pulpar reduction with age. The reasons for the pulpar size decrease can be found in age-related physiological pulp modifications. Young equine pulp tissue consists of odontoblasts, connective tissue, nerves, vessels and different cell types. Attachment of secondary dentine is detected and the vital cell number decreases with age [51]. As secondary dentine contains fewer protons, the pulp appears smaller in older equine cheek teeth in the MR images. In the CT images, pulpar tissue does not appears as well delineated as in the MRI scans. Therefore, pulpar existence, dimension and pathologies might not be detected as well with CT imaging as in the MRI scans. Finally, both imaging modalities, CT and MRI, seem to be inferior to histological examinations, which could be the reason for different pulpar dimensions found by Shaw et al. [54] and in imaging studies such as the current one.

In the present study, three pulps could not be measured in the MR images: one pulp was missing completely in each of the 09 s affected (Age group B: *n* = 1; Age group C: *n* = 2). All three teeth with one missing pulp in the MRI scans showed a higher attenuated or a gas spot-filled pulp in the CT scans. Missing pulps have been described by Gasse et al. [55], who carried out research on pulpar changes of the 07 s and 09 s in horses of different ages. Due to the expansion and proliferation of secondary dentine, the number of vital pulps is reduced in horses aged between 15 and 23 years. Fewer hydrogen atoms might result in less pulp detection in MRI scans and secondary dentine might be the explanation for higher attenuated pulps in the CT images of the current study.

Reasons for the absence of pulp in the MRI scans can also be found in pathogenic mechanisms that become visible comparing both imaging techniques. Whilst the MRI showed no causes for the absence of the pulp, CT revealed indications for infundibular and pulpar gas spots that can be interpreted as infundibular caries in the 09 s affecting the pulp horn. While Veraa et al. [56] and Bühler et al. [43] argued that infundibular changes often appear as a singular dental change in CT without significant relationship with pulpitis, Dacre et al. [57] describe infundibular changes which can cause and result in pulpitis, collapsing into the adjacent pulp. Inflammatory cells proliferate as a consequence of the pulpitis, which leads to varied pulp capillary blood flow, arteriovenous anastomoses and ischemic necrosis of the pulp [58]. Finally, this initiates the reduction of pulp size through the production of tertiary dentine [59], decreasing the pulpar visibility in MRI.

Other processes, such as dental trauma [43] or haematogenous pulp infection [47], can result in secondary pulpitis. In addition to pulp stones, histologically referred to as intra-pulpar calcified structures without tubular configuration [32], all these mechanisms can cause decreased vascularity of the pulp itself [60], pulpar infection and destruction, and its inability to be visualised in MR images.

It is described in other studies that the pulps sometimes underwent an occlusal insult, resulting in occlusal necrosis and the production of repairing tertiary dentine, but more apically the horns were vital [57]. This is the reason that all dorsally orientated MRI section planes should be assessed and reviewed for vital pulp tissue.

As a pathologically decreased or missing pulp in MRI does not always allow any conclusions regarding the aetiopathogenesis [17], evaluation of the adjacent structures complemented by CT imaging of bony and hard dental structures and occlusal surfaces is important.

Although all the horses examined appeared to be clinically healthy in terms of their teeth, the missing pulps (MRI) or infundibular gas spots (CT) might be an indicator for the start of dental pathology.

#### Distance between dental alveoli and the maxillary sinus and the infraorbital canal

Precise anatomical knowledge is essential for the evaluation of dental ascending infections and planning of surgical procedures. If a tooth with apical infection is located within the boundaries of the sinus cavity and induces a sinusitis, treatment of the sinus affected may be indicated [61].

Various declarations referring to the contact between teeth and paranasal sinuses due to inter-individual skull anatomy and age-related variances exist in the literature. While Hillmann [62] revealed that only the last three cheek teeth are in contact with the sinus floor, Dyce et al. [63] mentioned that the last premolar tooth’s alveolus is also in contact in young horses. In the current study, 94% of Triadan 08 and 09 showed contact with the maxillary sinus floor. The results obtained correspond to the increased risk of inducing secondary sinusitis in apically infected 08 s and 09 s that is described in literature [64].

Both the one-year-old teeth of Triadan 08 which did not show any contact with the sinus floor had direct contact to the IOC. If the IOC is in close contact with an infected tooth, local bony necrosis could occur due to facilitated expansion of proteolytic enzymes [65]. The distance between the alveoli of 08 s and 09 s and the IOC increased by an average of 1.9 mm per year with age. While a five-year-old tooth shows about 12 mm to the IOC, the distance is measured at 41 mm for a 20-year-old 08 or 09. Infraorbital nerve trauma, associated with neuritis and headshaking, is described as a complication of surgical tooth extraction and sinustomy [66]. Knowledge of the IOC’s position, as outlined above, can be essential for the prevention of these complications.

## Conclusions

The present study provides information about the dental and periodontal age-related morphology and its visibility via different imaging techniques in clinically healthy horses. Both 3 T MRI and CT provide a valuable tool for the visualisation and detection of dental and adjacent tissues. Both modalities complement each other, because MRI highlights soft dental and adjacent soft tissues and CT depicts hard dental and bony surrounding structures. The results aid in evaluating CT and MR images and in choosing the superior imaging modality. Although MRI is not applied as a routine diagnostic measure in dental pathologies, it provides some advantages that could be used for the detection of pulpar changes or before endodontic procedures. Future investigation is warranted to prove the opportunities of detecting and distinguishing pathological processes comparing MR and CT imaging. Knowledge about the age-related positional relations of the maxillary 08 s and 09 s and their adjacent structures expanded with imaging modalities (MRI, CT) might help to evaluate further clinical cases. False positive or negative results of dental pathologies can be avoided through optimal 3D imaging. Additionally, intra- or postoperative complications, such as IOC damage, can be prevented, as surgical planning is optimised.

## Abbreviations

3D: three dimensional
CPC: Common pulp chamber
CT: Computed tomography
e.g.: exempli gratia = for example
FOV: Field of view
IOC: Infraorbital canal
ISS: Inter-slice spacing
min: minutes
MPR: Multiplanar reconstruction
MR: Magnetic resonance
MRI: Magnetic resonance imaging
PDL: Periodontal ligament
PDw: Proton density weighted
pulp 1–5: pulp horn 1–5: P1-P5
ROI: Region of interest
ST: Slice thickness
STIR: Short-time-inversion-recovery
T1w: T1 weighted
T2w: T2 weighted
TE: Echo time
TR: Repetition time
WL: Window level
WW: Window width
